# Pneumopericardium induced by the radial force of a self-expandable metal stent in a patient with gastroesophageal junction adenocarcinoma: A case report

**DOI:** 10.1097/MD.0000000000047387

**Published:** 2026-02-06

**Authors:** Jun Su Lee, Jong Hee Sun, Jungmi Kim, Yook Kim, Seung-Myoung Son, Taek-Gu Lee, Hye Sook Han

**Affiliations:** a Department of Internal Medicine, Chungbuk National University Hospital, Chungbuk National University College of Medicine, Cheongju, Republic of Korea; b Department of Obstetrics and Gynecology, Chungbuk National University Hospital, Chungbuk National University College of Medicine, Cheongju, Republic of Korea; c Department of Radiology, Chungbuk National University Hospital, Chungbuk National University College of Medicine, Cheongju, Republic of Korea; d Department of Pathology, Chungbuk National University Hospital, Chungbuk National University College of Medicine, Cheongju, Republic of Korea; e Department of Surgery, Chungbuk National University Hospital, Chungbuk National University College of Medicine, Cheongju, Republic of Korea.

**Keywords:** adenocarcinoma, gastroesophageal junction, pneumopericardium, stent

## Abstract

**Rationale:**

Pneumopericardium is a rare clinical finding in cancer patients and is typically associated with direct tumor invasion or postoperative complications, particularly in intrathoracic malignancies, such as lung or esophageal cancer.

**Patient concerns:**

Herein, we report the case of a 58-year-old man with advanced gastroesophageal junction adenocarcinoma who developed pneumopericardium and pericarditis in the absence of tumor invasion.

**Diagnoses:**

The condition was presumed to result from the radial force exerted by a self-expandable metal stent placed for palliation of dysphagia. Computed tomography revealed the presence of air within the pericardial sac and a direct communication with the stent, without radiological evidence of tumor invasion into the pericardium.

**Interventions:**

Given the terminal nature of the disease and lack of response to palliative systemic therapy, the patient was managed conservatively.

**Outcomes:**

The patient succumbed to his illness 1.5 months later.

**Lessons:**

To the best of our knowledge, this is the 1st reported case of pneumopericardium induced by the radial force of a self-expandable metal stent in a patient with gastroesophageal junction adenocarcinoma, highlighting the importance of clinical vigilance for pneumopericardium, even in the absence of classic risk factors. Early recognition may guide appropriate management and inform prognostic discussions in patients receiving palliative care.

## 1. Introduction

Pneumopericardium, defined as the presence of air within the pericardial sac, is an uncommon but clinically significant condition that can precipitate life-threatening cardiac tamponade.^[1]^ This entity is most often encountered in the context of major thoracic trauma or iatrogenic injury during invasive cardiac and thoracic procedures.^[2]^ However, a broad range of other etiologies has been documented, including barotrauma from mechanical ventilation, direct communication between the pericardium and adjacent air-containing organs, gas-forming infections, and rare causes such as foreign body aspiration, illicit drug inhalation, or diaphragmatic hernia.^[3]^

Among cancer patients, pneumopericardium remains an exceedingly rare finding, typically occurring in the setting of intrathoracic malignancies. Most reported cancer-related cases have involved tumors of the lung or esophagus that erode into the pericardium (resulting in bronchopericardial or esophagopericardial fistulas) or have occurred as postoperative complications involving these anatomically adjacent organs.^[4,5]^ Indeed, only a handful of pneumopericardium cases associated with malignancy have been recorded in the past decade, and the majority were in patients with lung or esophageal cancer.^[6-9]^ These cases are notable for their rarity and often severe consequences, as air leak into the pericardial space in malignancy can rapidly progress to cardiac tamponade or purulent pericarditis if not promptly recognized.^[4]^

We report a rare case of pneumopericardium in a patient with gastroesophageal junction (GEJ) adenocarcinoma, which was not caused by tumor invasion but was attributable to the radial force of a self-expandable metal stent (SEMS).

## 2. Case presentation

A 58-year-old man with a known history of advanced GEJ adenocarcinoma presented to a secondary hospital with acute chest pain and dyspnea. The patient had been diagnosed with advanced GEJ adenocarcinoma with peritoneal seeding in January 2023. He had undergone first- and second-line palliative systemic therapy at another institution. To relieve progressive dysphagia caused by tumor-related obstruction, a fully covered SEMS (Bona^®^ stent, Standard SciTech Inc., Seoul, South Korea; 20 mm in diameter and 100 mm in length) was placed across the GEJ in July 2023. Following stent placement, he was transferred to our hospital for supportive care and subsequently received palliative third-line trastuzumab deruxtecan for 3 months. Two weeks prior to current presentation, disease progression was confirmed on response evaluation, and palliative fourth-line nivolumab was initiated.

**Figure 1.**
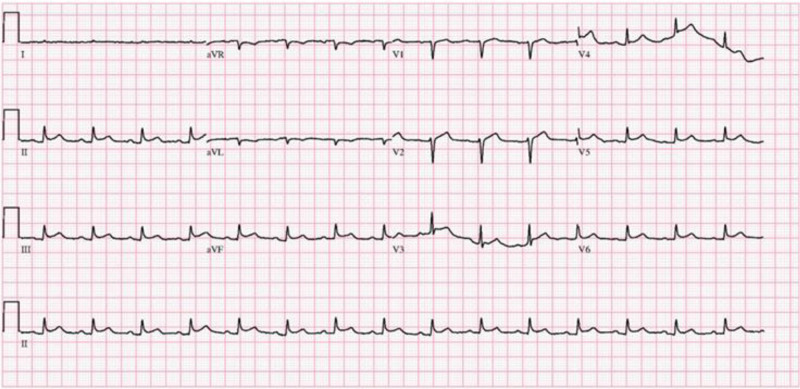
Electrocardiogram showing diffuse ST-segment elevations in the precordial leads, suggestive of pericarditis.

Upon arrival at our emergency department, the patient appeared acutely ill, presenting with hypotension despite prior fluid resuscitation and vasopressor support. His blood pressure was 73/51 mm Hg, and heart rate was 97 beats/min while receiving norepinephrine. Laboratory examinations revealed leukocytosis (white blood cell count, 14,550/μL), elevated C-reactive protein (18.65 mg/dL), and procalcitonin (3.81 ng/mL). Myocardial biomarkers were within normal ranges, with creatine kinase (21 U/L), creatine kinase-MB (0.549 ng/mL), and high-sensitivity troponin T (25 ng/L). Electrocardiogram revealed diffuse ST-segment elevations in the precordial leads, raising suspicion for acute pericarditis (Fig. 1).

**Figure 2.**
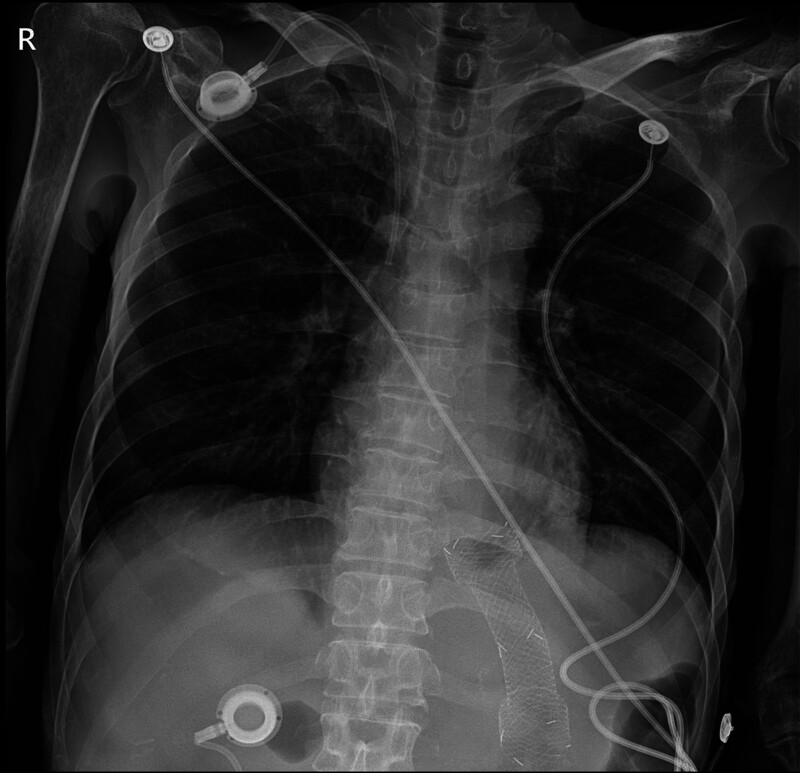
Chest radiography revealing no definitive evidence of pneumopericardium.

**Figure 3.**
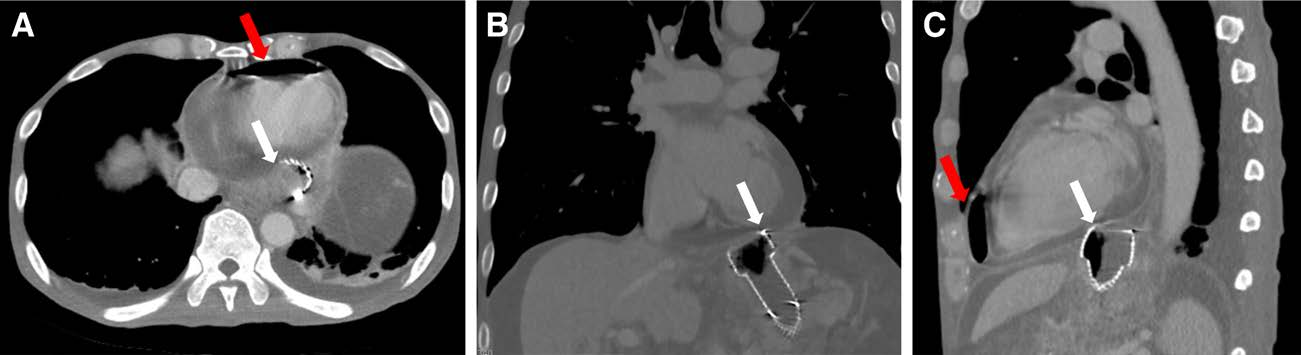
Chest CT images obtained at the initial presentation. (A) Axial view, (B) coronal view, and (C) sagittal view. Intrapericardial air is indicated by red arrows and white arrows indicate the esophageal stent closely abutting the pericardial contour. CT = computed tomography.

Chest radiography did not reveal any definitive evidence of free air around the cardiac silhouette (Fig. 2). However, computed tomography (CT) of the chest demonstrated multiple air pockets within the pericardial space, accompanied by a small to moderate pericardial effusion. Notably, a direct communication between the GEJ stent and the pericardial cavity was identified (Fig. 3). Transthoracic echocardiography (TTE) confirmed the presence of pericardial effusion without signs of hemodynamic compromise.

**Figure 4.**
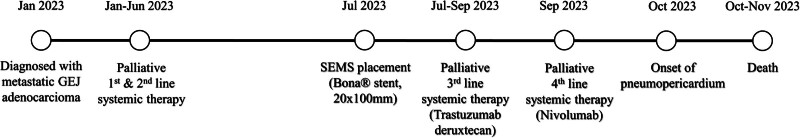
The timeline of the patient’s diagnosis and treatment course. GEJ = gastroesophageal junction, SEMS = self-expandable metal stent.

Although stent invasion into the pericardium was not definitively confirmed on TTE, no regional wall motion abnormalities were observed, and left ventricular systolic function was preserved. The patient was diagnosed with pneumopericardium with pericarditis, determined to be caused by the radial force from the SEMS. Empirical broad-spectrum antibiotics were initiated to treat the associated infection. Pericardiocentesis was not performed due to the relatively small amount of pericardial effusion and anatomically limited access. Additionally, stent repositioning or removal was also deferred given the high risk of further complications. Considering the patient’s poor prognosis, a decision was made to pursue conservative management. Follow-up CT imaging showed stable findings without further progression of pneumopericardium or effusion. However, the clinical status of the patient gradually declined due to disease progression, and he died 1.5 months after presentation. Figure 4 shows the timeline of the patient’s diagnosis and treatment course, including initial diagnosis, systemic therapies, stent placement, and the onset of pneumopericardium, followed by the clinical outcome.

The study protocol was approved by the Institutional Review Board of the Chungbuk National University Hospital (application no. 2025-10-028).

## 3. Discussion

Pneumopericardium, defined as the presence of air within the pericardial sac, is a rare clinical finding typically associated with trauma, mechanical ventilation, or invasive thoracic procedures.^[10-16]^ In cancer patients, it most commonly arises from direct tumor invasion or postoperative complications, particularly in intrathoracic malignancies such as esophageal or lung cancer.^[6-9]^ In contrast, pericardial involvement in GEJ adenocarcinoma is exceedingly rare; to the best of our knowledge, only 1 prior case has been reported in the literature.^[5]^ In the present case, pneumopericardium occurred in a patient with advanced GEJ adenocarcinoma without radiologic or echocardiographic evidence of direct tumor invasion into the pericardium. Notably, the temporal association with recent placement of a fully covered SEMS across the GEJ suggests a stent-related mechanism. Although a multifactorial interaction between nivolumab-induced immune effects and stent-related factors cannot be completely excluded, several findings favor a stent-driven etiology. The symptoms developed only 12 days after nivolumab initiation, chest CT revealed no evidence of tumor necrosis or cavitation, and the tumor exhibited malignant ascites with peritoneal seeding, low programmed death-ligand 1 combined positive score (CPS = 1), and a proficient mismatch repair status – all indicating minimal likelihood of an immune-mediated response. Moreover, CT images clearly demonstrated a direct communication between the stent tip and the pericardium. Taken together, these findings strongly suggest that localized ischemia or microperforation caused by the stent’s radial force was the primary mechanism leading to fistula formation and subsequent pneumopericardium. Hence, we hypothesize that the radial force of the SEMS may have induced localized ischemia or microperforation of adjacent structures, ultimately leading to fistula formation into the pericardial space.

Although stent-related pneumopericardium has been reported in other malignancies and even in benign conditions such as caustic ingestion,^[17]^ it remains an exceptionally rare occurrence. To the best of our knowledge, this is the first reported case of pneumopericardium associated with GEJ adenocarcinoma following endoscopic stent placement in the absence of tumor invasion. This case thus represents a unique combination of anatomical proximity, mechanical factors, and advanced disease.

Diagnosis of pneumopericardium can be challenging, particularly given the low sensitivity of plain chest radiographs. In this case, initial chest radiography failed to detect the presence of intrapericardial air. CT was instrumental in confirming the diagnosis by clearly visualizing air within the pericardial sac and identifying a fistulous tract between the stent and pericardium. Electrocardiographic findings of diffuse ST-segment elevation in the precordial leads, in conjunction with elevated inflammatory markers, supported the diagnosis of pericarditis. TTE further confirmed the presence of a small pericardial effusion without hemodynamic compromise.

Management of pneumopericardium should be according to the clinical context.^[5]^ In hemodynamically unstable patients or those with tension pneumopericardium, emergent pericardiocentesis may be required. However, in this case, the limited volume of pericardial effusion, high-risk anatomical location, and the patient’s poor overall prognosis due to refractory metastatic disease precluded invasive procedures such as pericardiocentesis and/or stent revision. Therefore, conservative management with close monitoring and supportive care was deemed appropriate.

## 4. Conclusions

This case highlights a rare but clinically significant complication of endoscopic stent placement in patients with advanced GEJ cancer. Although pneumopericardium is uncommon, it should be considered in the differential diagnosis when such patients present with acute cardiopulmonary symptoms, even in the absence of overt trauma or known signs of tumor invasion. Prompt recognition using appropriate imaging modalities, particularly CT, is essential for timely diagnosis. Additional case reports and studies are warranted to further elucidate the underlying pathophysiological mechanisms and to inform best practices for prevention, diagnosis, and management in similar cases.

## Author contributions

**Conceptualization:** Hye Sook Han.

**Data curation:** Jun Su Lee, Jong Hee Sun, Jungmi Kim, Yook Kim.

**Writing – original draft:** Jun Su Lee.

**Writing – review & editing:** Seung-Myoung Son, Taek-Gu Lee, Hye Sook Han.
